# Radioactivity and Hardness of Drinking Waters in Relation to Cancer Mortality Rates

**DOI:** 10.1038/bjc.1962.4

**Published:** 1962-03

**Authors:** R. C. Turner


### RADIOACTIVITY AND HARDNESS OF DRINKING WATERS IN RELATION TO CANCER MORTALITY RATES

R. C. TURNER

*From the Department of Physics, Institute of Cancer Research Royal Cancer Hospital, London, S.W.3.*

Received for publication January 13, 1962

A PREVIOUS paper by Turner, Radley and Mayneord (1961) presented the results of an investigation into the levels of naturally occurring alpha activity existing in the drinking waters of Britain. Attention was drawn to the fact that the daily intakes of radium 226 and its daughters, from this source alone, could vary by factors of up to 500 to 1 between individuals in different parts of the country. Earlier papers (Turner, Radley and Mayneord, 1958 ; and Mayneord, Radley and Turner, 1960) had shown that the daily intakes of radium from food could vary by factors of the same order, depending on the individual choice of diet. In the present investigation an attempt has been made to ascertain whether any differences can be observed between the mortality rates due to cancer of various sites in sections of the population whose drinking waters have widely different contents of natural radioactivity and of dissolved inorganic material.

The mortality data have been extracted from the Registrar General's Decennial Supplement, Area Mortality (1958) covering the four year period 1950–53 and based on the 1951 census. The Standardised Mortality Ratios (S.M.R.'s) provided by the Decennial Supplement have been used to compare mortality rates in local areas with that of England and Wales as a whole (S.M.R. = 100) for a number of causes of death.

The natural alpha activities of the drinking waters are those previously reported (Turner *et al.*, 1961) for 71 supplies supplemented by data for an additional 30 sources measured more recently. During the past year, serial measurements have been made on the residues resulting from evaporation of the original water samples, special attention being given to those derived from Wales. Fractional increases of alpha activity, presumably due to the growth of polonium 210, were observed in a few cases, but the majority of the specimens remained unchanged in activity throughout the period. In none of the water samples could evidence be found of the presence of lead 210 at a level of activity comparable with that due to radium 226 and its daughters.

### *Water activity and area mortality rates*

The selection of two regions of the country having drinking waters containing widely different amounts of natural radioactivity, presents no problem, since it has been shown (Turner *et al.*, 1961) that, apart from certain spa waters, the highest levels are to be found in the drinking water supplies of Cornwall. On the other hand, many of the waters supplied to the population of Wales contain extremely low levels of natural radioactivity, barely measureable even with highly sensitive modern techniques.

The maximum level of natural alpha activity so far observed in drinking water in Cornwall is 10 micromicrocuries per litre due to long lived radium 226 and its daughters, together with 10,000 to 20,000 micromicrocuries per litre of dissolved radon 222 and its alpha, beta and gamma emitting decay products. It is probable that waters containing levels of natural radioactivity at least as high, have been consumed by the population of that county for a very considerable time. A number of the wells, springs and streams which, before the extension of piped supplies, must have contributed to the available drinking water are known to contain still higher levels of natural activity (Peacock, 1961). If we take the observed level of activity and the estimated number of consumers of each of the principal water supplies in Cornwall, the calculated weighted mean value of radioactivity for the drinking water of the county is approximately 1·8 micromicrocurie per litre due to long lived radium and its daughters and 3000–4000 micromicrocuries per litre due to relatively short lived dissolved radon gas and its decay products.

For the purposes of comparison, the three Welsh counties of Caernarvon, Merioneth and Cardigan together comprise an area where the drinking waters have very low natural activity, the weighted mean value of long lived activity being only of the order of 0·05 micromicrocurie per litre. In other words, the mean daily intake of radium 226 and its daughters, from drinking water, is some 36 times, and of radon and its daughters more nearly 1000 times higher in Cornwall than in the three selected Welsh counties. Both areas are predominantly rural, possess extensive coast line and have a sufficiently large population for mortality rates over a four year period to be reasonably defined for a number of diseases.

Fig. 1 gives the Standardised Mortality Ratios (S.M.R.'s) in the two areas for males of all ages, for a number of causes, including all the types of cancer listed in the Registrar General's (1958) Decennial Supplement covering the period 1950 to 1953. Fig. 2 gives the corresponding pattern of female mortalities. The total number of deaths from each cause upon which each particular S.M.R. is based is indicated on the diagrams.

It is evident from Fig. 1 that, with the exception of diabetes mellitus, the male population of Cornwall shows no surplus mortality during the period from any of the causes listed. On the contrary, there appeared to be a deficiency of carcinoma trachea, lung and bronchus and of bronchitis and pneumonia. For deaths from all causes the S.M.R. = 93. In the Welsh area, on the other hand, there was a large surplus of gastric cancer and of respiratory tuberculosis, the S.M.R. for deaths due to all causes being 104.

In Fig. 2 the female population of Cornwall appear to show a surplus of deaths due to diabetes mellitus, although not so pronounced as in the males, together with surplus mortality from carcinoma uterus and a possible slight excess of gastric cancer. Bronchitis and pneumonia were significantly below the average for England and Wales as also was cancer of the trachea, lung and bronchus. The S.M.R. for deaths from all causes is given as 103.

In the Welsh counties the female population showed a considerable excess of gastric cancer together with a surplus of deaths from vascular lesions affecting the central nervous system. As in the case of Cornwall, the area showed deficiencies of both bronchitis and pneumonia. The S.M.R. for all causes of death is given as 107.

The small numbers of deaths from leukaemia occurring in each sex do not permit separate comparisons of the mortality rates due to this cause in the two areas. If we combine the figures for males and females, we have 71 observed deaths in

Cornwall compared with 66 expected deaths. There is no evidence therefore of any surplus mortality from leukaemia in Cornwall during the period under observation.

The excess of deaths from diabetes mellitus in both sexes in Cornwall is regarded as worthy of note and of future investigation, but will not be discussed in the present paper.

FIG. 1.—Comparison of the S.M.R.'s for a number of diseases among males in Cornwall and Wales.

Whilst we appreciate that Fig. 1 and 2 deal only with mortality rates and for obvious reasons take no account of relative morbidities, it is clear from the mortality rates, that in spite of the relatively high contents of radium 226 and of radon 222 in the drinking waters of Cornwall, there is no evidence of any excess cancer of the breast, uterus, trachea, lung, bronchus, stomach, or of leukaemia occurring in Cornwall, compared with the three Welsh counties. On the contrary, although the Welsh area has drinking waters having extremely low natural radioactivity, a considerable excess of gastric cancer occurs in both sexes. This surplus gastric cancer mortality has been observed for many years and has been reported by a number of investigators (Stocks, 1947 ; Stocks and Davies, 1960 ; Millar, 1961 ; Howe, 1959).

Fig. 2.—Comparison of the S.M.R.'s for a number of diseases among females in Cornwall and Wales.

Further evidence that this surplus mortality is very unlikely to be due to natural alpha activity in the drinking water is provided by Fig. 3 in which the S.M.R.'s for gastric cancer in the two sexes have been plotted against the long lived alpha activity of the drinking waters for a number of counties and county boroughs of England and Wales. Every county borough in which the natural radioactivity of the drinking water has been measured (Turner *et al.*, 1961), and for which the Registrar General provides the S.M.R. for gastric cancer, has been included in the diagram. In cases where a county borough derives its water from more than one source, a calculated weighted mean activity has been used based on the relative bulk contributions made by the various sources. These latter data have been obtained partly from the Water Engineer's Handbook (1959) and partly from information supplied by the Ministry of Housing and Local Government during the preparation of a previous paper (Turner *et al.*, 1961). In cases where the activities of a sufficient number of the different supplies available in a particular county have been measured, a weighted mean value of activity has been calculated, based on the number of consumers of each individual supply, and the county has been included in Fig. 3.

FIG. 3.—Distribution of the S.M.R.'s for gastric cancer in relation to the radioactivity of drinking water, 1950–53. Total population represented = 18 millions.

✦ Counties of Caernarvon, Merioneth and Cardigan.

● County.

○ County borough.

The total population represented in the diagram is approximately 18 millions and the radium contents of the drinking waters extend over a range of almost 50 to 1. The county boroughs and counties depicted in Fig. 3 as having the higher values of long lived activity in their water supplies (greater than approximately 0·4 micromicrocurie per litre) have been observed to have relatively high levels of radon and its daughters, up to several hundred micromicrocuries per litre, also present in the water. This seems to be the case when the water is derived from boreholes in geological strata other than chalk and does not happen to be subjected to surface storage for a period long compared with the 3·8 day radioactive half life of radon 222. Conversely the county boroughs and counties with the lowest levels of long lived activity in their water supplies are frequently areas deriving their water from surface drainage. Such waters have been observed to contain only very low radioactivity due to dissolved radon.

Fig. 3 takes no account of this fact and only deals with the long lived activity present in the waters.

It will be seen that there is no relationship apparent between the mortality rates due to gastric cancer and the radium contents of the drinking waters in the various centres of population.

The combined Welsh counties of Caernarvon, Merioneth and Cardigan remain outstanding with the highest mortality rate and the lowest activity drinking water, whereas the county borough with long lived activity some 50 times higher in its water (plus, incidentally, 100 to 200 micromicrocuries per litre of radon and its daughters) shows an S.M.R. = 100 in both sexes.

Whether we compare the mortality rates for cancer of different sites or for other causes of death, in two areas having very different levels of natural radioactivity in their drinking water, or whether we consider the distribution of gastric cancer in terms of such water activity, we are forced to the same conclusion.

Within the not inconsiderable range of natural activities present in the drinking waters of England and Wales, there is no evidence in the Registrar General's (1958) returns for the period 1950–53, that even the highest observed contents of radium or of radon are in any way responsible for increased mortality due to cancer of the stomach or of any of the other sites considered above.

*Water hardness and mortality rates*

During a previous investigation into the natural radioactivities of drinking waters, the total amount of dissolved inorganic material present in each water sample was determined (Turner *et al.*, 1961). It was observed at the time, that the specimens which originated in North Wales yielded very small amounts of residue after evaporation, and that by Water Engineer's standards they were very soft waters, often containing only a few parts per million of inorganic material. On the other hand, the drinking waters examined from the eastern and southern regions of England were, by the same standards, usually hard or very hard, with inorganic contents ranging up to 400 parts per million.

It is of interest to compare the mortality rates from gastric cancer exhibited by different sections of the population, in terms of the hardness of their drinking waters.

The total hardness and the approximate number of consumers of each of the 1000 principal water supplies of England and Wales have been extracted from the Water Engineer's Handbook (1959). For comparison of mortality rates in different counties, the total hardness of each principal supply available in a given county has been weighted with the estimated number of consumers and an overall weighted mean figure derived for the total hardness of the drinking water in that county.

For comparison of mortality rates in county boroughs having more than one source of water, a similar procedure has been adopted, based on the relative magnitude of the bulk contributions made by the different supplies.

These calculations have involved a considerable amount of labour, but it is thought that the weighted mean values of water hardness thus derived are the best estimates that can be made on the existing data. The total number of water supplies included in these calculations represents the drinking waters consumed by more than 90 per cent of the population of England and Wales.

Fig. 4 shows the result of plotting the S.M.R. for gastric cancer in males of all ages against the weighted mean total hardness of the drinking water, for the counties of England and Wales. All counties are included in the diagram with the exception of Radnor, Rutland, Westmorland, Soke of Peterborough and Hunting-

FIG. 4.—County distributions of the S.M.R.'s for gastric cancer among males of all ages in terms of water hardness, 1950–53. Counties of England and Wales.

● English county.

○ Welsh county.

| | Total hardness | | |
|---|---|---|---|
| | <50 p.p.m. | <100 p.p.m. | >100 p.p.m. |
| Actual deaths | 1,650 | 7,029 | 14,651 |
| Expected deaths | 1,427 | 6,368 | 16,280 |
| S.M.R. | 116 | 111 | 90 |

don, whose relatively small populations did not permit reasonable assessment of the S.M.R. over the four year period 1950–53 covered by the Registrar General's (1958) Decennial Supplement. Fig. 5 gives the corresponding diagram for females of all ages. In both sexes the S.M.R. appears to rise sharply above the national average of 100 in counties with increasingly soft water supplies. On the other hand, the large majority of counties with water supplies containing more than about 140

parts per million of inorganic matter showed significant deficiencies of gastric cancer in both sexes during the period.

The data contained in the two diagrams are summarised in Table I.

TABLE I

| County Drinking water total hardness parts per million | Males | | Females | | S.M.R. | |
|---|---|---|---|---|---|---|
| | Actual deaths | Expected deaths | Actual deaths | Expected deaths | M. | F. |
| Less than 50 | 1,650 | 1,427 | 1,299 | 1,091 | 116 | 119 |
| „ „ 100 | 7,029 | 6,368 | 5,561 | 4,802 | 111 | 116 |
| Exceeding 100 | 14,651 | 16,280 | 11,493 | 12,830 | 90 | 89 |
| „ 200 | 10,842 | 12,401 | 8,647 | 10,057 | 87 | 86 |

FIG. 5.—County distributions of the S.M.R.'s for gastric cancer among females of all ages in terms of water hardness, 1950–53. Counties of England and Wales.

● English county.

○ Welsh county.

| | Total hardness | | |
|---|---|---|---|
| | <50 p.p.m. | <100 p.p.m. | >100 p.p.m. |
| Actual deaths | 1,299 | 5,561 | 11,493 |
| Expected deaths | 1,091 | 4,802 | 12,830 |
| S.M.R. | 119 | 116 | 89 |

It will be observed that counties whose drinking waters contain less than 50 parts per million of dissolved mineral matter show an S.M.R. some 33 per cent higher for males and 38 per cent higher for females than counties which have hard waters, containing more than 200 parts per million of inorganic matter. If instead of using the S.M.R.'s for males and females of all ages, we consider only the age group 45 to 64 years, the general shape of the patterns in Fig. 4 and 5 do not

FIG. 6.—County borough distributions of the S.M.R.'s for gastric cancer among males of all ages in terms of water hardness, 1950–53.
● English county borough.
○ Welsh county borough.

significantly change, apart from a few more counties having to be omitted by virtue of the smaller numbers of deaths then involved. Furthermore, if we derive and plot the weighted mean values of the temporary instead of the total hardness of the waters, the resultant patterns for all ages and for the age group 45 to 64 years remains substantially unchanged from Fig. 4 and 5.

It is evident from the two diagrams that the counties exhibiting surplus mortality from gastric cancer and having at the same time soft drinking waters are mostly counties of Wales. One might therefore argue from Fig. 4 and 5 that the surplus mortality in these areas is a reflection of a characteristic of Welsh populations and is only coincidentally related to water softness. That this is not the case will be seen from Fig. 6 and 7, in which the individual S.M.R.'s for the majority of the county boroughs listed in the Decennial Supplement (Registrar General, 1958) are plotted against the values of total hardness of their drinking waters. Of the 82 county boroughs only 4 are in Wales and these together contribute only 5 per cent of the total gastric cancer deaths represented in Fig. 6 and 7. A similar trend to-

FIG. 7.—County borough distributions of the S.M.R.'s for gastric cancer among females of all ages in terms of water hardness, 1950–53.

● English county borough.
○ Welsh county borough.

wards higher gastric cancer mortality with increasing softness of drinking water is displayed by county boroughs as was observed in the counties and again the patterns do not significantly change if the age group 45 to 64 years is substituted for " all ages ". Table II summarises the data contained in Fig. 6 and 7 and indicates approximately 25 per cent higher mortality in both sexes in county boroughs with soft water supplies than in those with more than 200 parts per million of dissolved mineral matter.

Evidently this phenomenon is not confined to Welsh populations and further evidence of this fact emerges from consideration of the mortality rates for gastric

TABLE II

| County Borough Drinking water total hardness parts per million | Males | | Females | | S.M.R. | |
|---|---|---|---|---|---|---|
| | Actual deaths | Expected deaths | Actual deaths | Expected deaths | M. | F. |
| Less than 50 | 3898 | 3227 | 3216 | 2718 | 121 | 119 |
| „ „ 100 | 4972 | 4218 | 4070 | 3528 | 118 | 116 |
| Exceeding 100 | 3796 | 3845 | 3021 | 3173 | 99 | 95 |
| „ 200 | 2230 | 2317 | 1811 | 1917 | 96 | 95 |

cancer in the county of Yorkshire. The Decennial Supplement (Registrar General, 1958) provides separate mortality figures for the East, North and West Ridings of that county and it so happens that the water supplies of the three areas have very different values of total hardness. A calculated weighted mean value of total hardness, based on the number of consumers of each principal water supply, has been derived for each of the Ridings.

These weighted mean figures together with the mortality data for gastric cancer in the three areas are given in Table III.

TABLE III

| Area | Total hardness Parts per million | Males all ages | | | Females all ages | | |
|---|---|---|---|---|---|---|---|
| | | Actual deaths | Expected deaths | S.M.R. | Actual deaths | Expected deaths | S.M.R. |
| East Riding | ~250 | 133 | 175 | 76 | 117 | 133 | 88 |
| North Riding | ~150 | 261 | 281 | 93 | 190 | 214 | 89 |
| West Riding | ~45 | 1252 | 1180 | 106 | 978 | 873 | 112 |
| Less than 100 p.p.m. | | 1252 | 1180 | 106 | 978 | 873 | 112 |
| Exceeding 100 p.p.m. | | 394 | 456 | 86 | 307 | 347 | 89 |

It is clear from the table that the trend towards higher mortality in the soft water areas is still maintained in both sexes even if we consider the distribution of gastric cancer within just a single county. The question arises whether a similar trend towards higher mortality rates in areas of increasingly soft drinking waters is displayed by cancer of any other site.

Fig. 8 gives the distribution of the S.M.R.'s for cancer of the breast and uterus respectively, in the counties of England and Wales, plotted against the total water hardness. The greater scatter of the points in the diagram for cancer of the uterus is presumed due in part to the relatively smaller number of deaths from this cause in each county, compared with the corresponding figures for breast cancer. In neither case does the distribution bear any resemblance to the pattern obtained for gastric cancer in females in Fig. 5.

Fig. 9 gives the county distribution for cancer of the trachea, lung and bronchus in the two sexes. Both patterns tend to be dominated by the high mortality rates appearing in London and Middlesex, but in neither case does the distribution resemble that shown for gastric cancer in Fig. 4 and 5. Evidently the trend towards higher mortality rates with increasing softness of water is not shown by cancer of any of these sites.

In order to investigate this point in greater detail, 71 of the county boroughs of England together with the 29 metropolitan boroughs have been divided into two groups. Group 1 consists only of the 33 county boroughs with soft drinking waters ranging in total hardness from 0 to 100 parts per million, while Group 2 includes the 38 county boroughs and 29 metropolitan boroughs with hard drinking waters ranging upwards from 100 to 400 parts per million of dissolved mineral matter. The populations of the two groups are not very different and it will be observed that the Welsh population, with its very soft drinking waters, has been deliberately excluded. The division into only two groups tends to minimise any differences which may exist between mortality rates in areas of differing water hardness, such as that indicated by Fig. 4 and 5 for example, and one might expect to distinguish only the gross differences, if any, between the groups.

FIG. 8.—County distributions of the S.M.R.'s for cancer of the breast and uterus, all ages, in terms of water hardness. England and Wales, 1950–53.

● English county.

○ Welsh county.

Table IV gives the number of actual and of expected deaths, in males of all ages, for a number of different causes, in the two groups of county boroughs, while Table V gives the data for females of all ages. The figures apply to the period 1950–53 and have been extracted from the Decennial Supplement (Registrar General, 1958).

It will be observed that, in spite of the exclusion of the Welsh population with their very soft drinking waters, the soft water areas in Group 1 still show a surplus mortality from gastric cancer in both sexes, the surplus being rather more pronounced in females.

FIG. 9.—County distributions of the S.M.R.'s for cancer of the trachea, lung and bronchus in both sexes, all ages, in terms of water hardness. England and Wales, 1950–53.

● English county.

○ Welsh county.

TABLE IV.—*Males All Ages*

| Cause | Group 1 33 County boroughs Total hardness less than 100 p.p.m. Observed deaths | Expected deaths | S.M.R. | Group 2 38 County boroughs and 29 metropolitan boroughs Total hardness 100 to 400 p.p.m. Observed deaths | Expected deaths | S.M.R. |
|---|---|---|---|---|---|---|
| Cancer stomach | 5,341 | 4,677 | 115 | 6,289 | 6,268 | 100 |
| Leukaemia and aleukaemia | 633 | 627 | 101 | 895 | 821 | 109 |
| Cancer trachea, lung and bronchus | 8,452 | 6,842 | 124 | 11,460 | 9,013 | 127 |
| Diabetes mellitus | 672 | 650 | 103 | 919 | 875 | 105 |
| Vascular lesions affecting C.N.S. | 18,377 | 16,211 | 113 | 20,427 | 22,093 | 92 |
| Atherosclerosis and coronary disease | 23,718 | 21,636 | 110 | 29,679 | 29,101 | 102 |
| Respiratory tuberculosis | 5,657 | 4,283 | 132 | 7,076 | 5,589 | 127 |

TABLE V.—*Females All Ages*

| Cause | Group 1 33 County boroughs total hardness less than 100 p.p.m. Observed deaths | Expected deaths | S.M.R. | Group 2 38 County Boroughs and 29 metropolitan boroughs total hardness 100 to 400 p.p.m. Observed deaths | Expected deaths | S.M.R. |
|---|---|---|---|---|---|---|
| Cancer breast | 4,785 | 4,908 | 97 | 6,720 | 6,587 | 102 |
| Cancer uterus | 2,751 | 2,471 | 111 | 3,354 | 3,303 | 102 |
| Cancer stomach | 3,698 | 3,225 | 115 | 4,937 | 5,220 | 94 |
| Leukaemia and aleukaemia | 552 | 570 | 97 | 789 | 753 | 105 |
| Cancer trachea, lung and bronchus | 1,459 | 1,300 | 112 | 2,114 | 1,759 | 120 |
| Diabetes mellitus | 1,478 | 1,375 | 107 | 1,794 | 1,899 | 94 |
| Vascular lesions affecting the C.N.S. | 24,797 | 23,350 | 107 | 29,381 | 32,542 | 90 |
| Atherosclerosis and coronary disease | 13,115 | 12,696 | 103 | 18,151 | 18,029 | 101 |

On the other hand no statistically significant difference appears between the S.M.R.'s for cancer of the trachea, lung and bronchus in the two groups of males or between the two groups of females. In the case of cancer of the breast and of cancer of the uterus, there is also no apparent difference between the S.M.R.'s for the two groups.

These findings are compatible with the county distributions shown in Fig. 8 and 9 for cancers of the three sites.

No difference appears in the S.M.R.'s for respiratory tuberculosis or for diabetes mellitus in the two groups of males. The female population of the soft water areas of Group 1 show surplus mortality (S.M.R. 107) due to diabetes mellitus compared with those of Group 2 (S.M.R. 94). This latter fact is worthy of note, since Tables IV and V do not include the population of Wales with its relatively very soft drinking waters. It is interesting to observe that the S.M.R.'s for diabetes mellitus in the whole of Wales during the same period showed surplus mortality in females (S.M.R. 121) but not in males (S.M.R. 97). This surplus mortality due to diabetes mellitus among the female population of regions having soft drinking waters appears not unlike the findings above with regard to gastric cancer, except that the latter shows surplus mortality in both sexes in the soft water areas.

In the case of vascular lesions affecting the central nervous system, the mortality rates appear significantly higher in both sexes in Group 1 (male S.M.R. 113, female S.M.R. 107) than in the harder water areas of Group 2 (male S.M.R. 92, female S.M.R. 90). A similar finding has been reported by other investigators (Morris, Crawford and Heady, 1961 ; Schroeder, 1960). Atherosclerotic heart and coronary disease, on the other hand, shows a slight surplus mortality only among males in the soft water areas.

Earlier in the present paper, during the comparison of mortality rates in two areas having widely different water activities, it was difficult to discuss the rates for leukaemia because of the small number of deaths from this cause in the two regions during the four year period. Tables IV and V refer to considerably larger populations and it might be convenient, at this point, to consider the mortality rates due to this type of cancer.

The 100 county boroughs comprising Groups 1 and 2 do not include the population of Cornwall, which has no county borough listed in the Decennial Supplement

(Registrar General, 1958). We have already seen that the water supplies in Cornwall have higher levels of natural radioactivity than any other waters so far examined in England and Wales. Furthermore, they are anomalous in the sense that their high activities are not the result of their having high contents of dissolved inorganic material, but are due to the relatively high specific activity of the often small amounts of mineral matter present in them. Over the rest of the country the drinking waters containing the largest amounts of dissolved inorganic material, i.e. the hardest waters, generally are found to have the highest contents of natural radioactivity (Turner *et al.*, 1961). The fact that Cornwall is not included in the data presented in Table IV and V means, therefore, that the soft waters of Group 1 are the waters with the lowest levels of long lived natural activity. The harder waters of Group 2, with their greater mineral contents, are estimated to have a mean value of long lived radioactivity some 20 times higher than the mean value present in those of Group 1. For dissolved radon and its decay products, the mean level is of the order of 100 times greater in the drinking waters of Group 2.

The slight differences between the S.M.R.'s for leukaemia in the two groups, indicated by Tables IV and V, are not statistically significant.

One other type of cancer, sometimes associated with radiation damage, is the primary bone tumour. The number of deaths occurring in the population of England and Wales due to this cause is approximately 200 per year, and the Decennial Supplement (Registrar General, 1958) provides no information on their geographical distribution. A detailed investigation into the incidence of such tumours has, recently been reported (Mackenzie, Court Brown, Doll and Sissons, 1961 ; Court Brown, Doll and Heasman, 1961) and we have extracted the data on their distribution in England and Wales during the period 1951–53. Fig. 10 shows the result of plotting the S.M.R.'s for primary bone tumours for a number of counties and county boroughs, in terms of the total hardness of their drinking waters. The diagram provides no evidence of increased mortality from such tumours in areas having the harder waters. There appears to be a trend in the opposite direction, even more impressive than that indicated in Fig. 4 and 5 for gastric cancer.

If we divide the data into two groups, the S.M.R. for the soft water regions (less than 100 parts per million) is 114 compared with 91 for the harder water areas (100 to 400 parts per million). Cornwall is separately identified on the diagram and shows no surplus mortality from primary bone tumours. Using a previous argument, if we exclude Cornwall, then the scale of increasing water hardness which forms the abscissa of Fig. 10 may be regarded as a scale of increasing natural radioactive content of the drinking waters. The range of radium contents covered by the points in the diagram is again approximately 20 to 1, and the range of radon levels of the order of 100 to 1.

There is no evidence of increased mortality due to primary bone tumours in the populations consuming drinking water containing the highest levels of natural radioactivity.

### DISCUSSION

A number of investigators (Stocks, 1947 ; Stocks and Davies, 1960 ; Millar, 1961 ; Allen-Price, 1960) have suggested that surplus mortality rates due to gastric or other cancer exhibited by particular regions of England and Wales might be a result of abnormally high levels of natural radioactivity in the soils, subsequently appearing in the drinking waters and possibly in locally grown foods in the areas concerned. The data presented in this paper do not provide any evidence of such an association between cancer mortality rates and the natural radioactivities of drinking waters in different parts of this country. The mortality figures for Cornwall during the period 1950–53 show no surplus leukaemia, primary tumours of bone, or cancer of other sites in spite of the relatively high levels of natural radioactivity which have been present for a considerable time in the drinking water

FIG. 10.—Distribution of the S.M.R.'s for primary tumours of bone in terms of water hardness. England and Wales, 1951–53.

| | <100 p.p.m. | >100 p.p.m. |
|---|---|---|
| Actual deaths | 265·8 | 331·7 |
| Expected deaths | 233·9 | 363·8 |
| S.M.R. | 114 | 91 |

supplies of that county. On the other hand, a number of Welsh Counties, whose drinking waters are known to contain very low levels of natural activity, show considerable surplus mortality due to gastric cancer in both sexes. This phenomenon is not confined to Welsh populations, nor can it be a result of ingesting abnormally high levels of natural radioactivity in drinking water.

The regions of England and Wales which show significantly excess mortality from gastric cancer tend to be those with soft or very soft drinking waters and we have seen that, with the exception of Cornwall, these are the waters with the lowest levels of natural radioactivity. This pattern of increasing gastric cancer mortality with decreasing amount of inorganic material in the drinking water appears to be maintained regardless of whether the analysis is based on selected groups of either sex, in counties, county boroughs or even within a single county such as Yorkshire. It is interesting that surplus mortality due to diabetes mellitus among females also appears to be a feature of the soft water regions, and a similar trend occurs in the mortality rates due to vascular lesions affecting the central nervous system in both sexes. These apparent anomalies are emphasised by the fact that the mortality rates due to cancer of other sites, such as the trachea, lung, bronchus, breast and uterus, and those for leukaemia, do not follow this pattern of distribution.

The composition of the inorganic matter present in any particular drinking water is determined by a number of factors, including the nature of the soil, or the geological formations over or through which the water has passed and the degree of acidity possessed by the water before treatment. Examination of the underlying geological strata does not disclose any obvious feature common to the areas of excess gastric cancer mortality. The strata range in age from the ancient Cambrian and Ordovician formations encountered in Wales to the more recent Carboniferous and New Red Sandstone areas of Lancashire and Yorkshire. It is certainly true that many of the softer waters derive from peaty moorland areas and are relatively acid, preceding adjustment of their pH before they are supplied for human consumption. If variations occur in the efficiency with which the pH adjustment is effected in different regions, it would presumably be possible for several water supplies to differ in their final quality, even if they were originally derived from a common catchment area.

Remarkably little knowledge exists concerning the relative amounts of the numerous stable elements which may be present in drinking water. Certainly no information is available, at present, concerning the physiological effects of consuming, over long periods of time, water containing known quantities of particular elements. The presence of a number of these substances in water may well be essential for the continued well-being of biological systems, including the human, despite the fact that their total mass may represent an inorganic content of only 1 or 2 parts per million. Even the softest waters could therefore contain a number of such elements at concentrations which, in the light of future knowledge, would be regarded as abnormally high. On the other hand, future investigations may indicate that, from a physiological point of view, such waters are deficient in certain essential elements.

Still less information is available concerning the organic substances occurring in drinking waters. Precise and effective tests, specifications and treatment processes are directed towards the removal of certain types of pathogenic organism, but with this exception, little attention has been given to the identification and quantitative assessment of the various organic constituents of drinking waters. The softer waters, originally acid in character which result from surface drainage of moorland areas, could well contain amounts of a number of organic substances greatly in excess of the levels present in the harder waters derived from the deep wells and boreholes of other parts of the country.

Evidently a standard of water quality, which is related to human physiological requirements, cannot exist until the physiological roles played by the various trace elements and organic substances present in water are more clearly defined. These are necessarily long term investigations, but in the meantime it is likely that valuable indications would emerge from detailed study of the trace elements and the organic substances present in drinking waters in different parts of the world. A beginning has already been made in the United States of America where Durum

(1960) has reported data on the relative amounts of 25 different elements observed in 15 major rivers of North America.

Using activation analysis and spectroscopic techniques, this Department has commenced investigations aimed at defining the quantities of individual stable elements occurring in the drinking water supplies of Great Britain. The need for parallel studies of the organic substances appearing in drinking water, is no less real and becomes increasingly urgent.

It seems probable that consideration of mortality rates in relation to this type of data, rather than to radioactivity, might well further our understanding of regional differences in the rates shown by a number of diseases, including gastric cancer.

### SUMMARY

Study of mortality rates for cancer of different sites and for a number of other diseases does not reveal any correlation between area mortality and the widely different amounts of radium 226 and its daughter products known to be present in the drinking waters available in England and Wales. The areas of Wales which show surplus mortality from gastric cancer possess drinking water supplies with very low contents of natural radioactivity.

Surplus gastric cancer tends to appear in regions having soft drinking waters derived from surface drainage, and often having a low pH at their source. This pattern appears to be maintained regardless of whether the analysis is based on differences between counties, county boroughs or the parts of a single large county such as Yorkshire.

Surplus mortality rates, due to diabetes mellitus among females and to vascular lesions affecting the central nervous system in both sexes, also appear in the soft water regions of England and Wales. This trend is not displayed by cancer of other sites nor does it appear in the mortality data for a number of other diseases.

Attention is drawn to the almost complete lack of knowledge existing throughout the world concerning the trace elements and the organic constituents of drinking water.

In conclusion, I would like to express my gratitude to Professor W. V. Mayneord, the Director of this Department, for his encouragement throughout this investigation.

### REFERENCES

ALLEN-PRICE, E. D. (1960) *Lancet*, i, 1235.

COURT BROWN, W. M., DOLL, R. and HEASMAN, M. A.—(1961) *Brit. J. prev. soc. Med.*, **15**, 167.

DURUM, W. H.—(1960) 'Proceedings of Conference on Physiological Aspects of Water Quality.' Washington, D.C., p. 51.

HOWE, G. M.—(1959) *Brit. J. prev. soc. Med.*, **13**, 204.

MACKENZIE, A., COURT BROWN, W. M., DOLL, R. AND SISSONS, H. A.—(1961) *Brit. med. J.*, i, 1782.

MAYNEORD, W. V., RADLEY, J. M. AND TURNER, R. C.—(1960) *Advanc. Sci., Lond.*, **64**, 363.

MILLAR, I. B.—(1961) *Brit. J. Cancer*, **15**, 175.

MORRIS, J. N., CRAWFORD, M. D. and HEADY, J. A.—(1961) *Lancet*, i, 860.
PEACOCK, J. D.—(1961) *Nature, Lond.*, **191**, 1189.
REGISTRAR GENERAL—(1958) 'Decennial Supplement—England and Wales. Area Mortality.' London. (H.M. Stationery Office.)
SCHROEDER, H. A.—(1960) *J. Amer. med. Ass.*, **172**, 1902.
STOCKS, P.—(1947) 'Regional and Local Differences in Cancer Death Rates.' London (H.M. Stationery Office).
*Idem* AND DAVIES, R. I.—(1960) *Brit. J. Cancer*, **14**, 1.
TURNER, R. C., RADLEY, J. M. AND MAYNEORD, W. V.—(1958) *Health Phys.*, **1**, 368.—(1961) *Nature, Lond.*, **189**, 348.


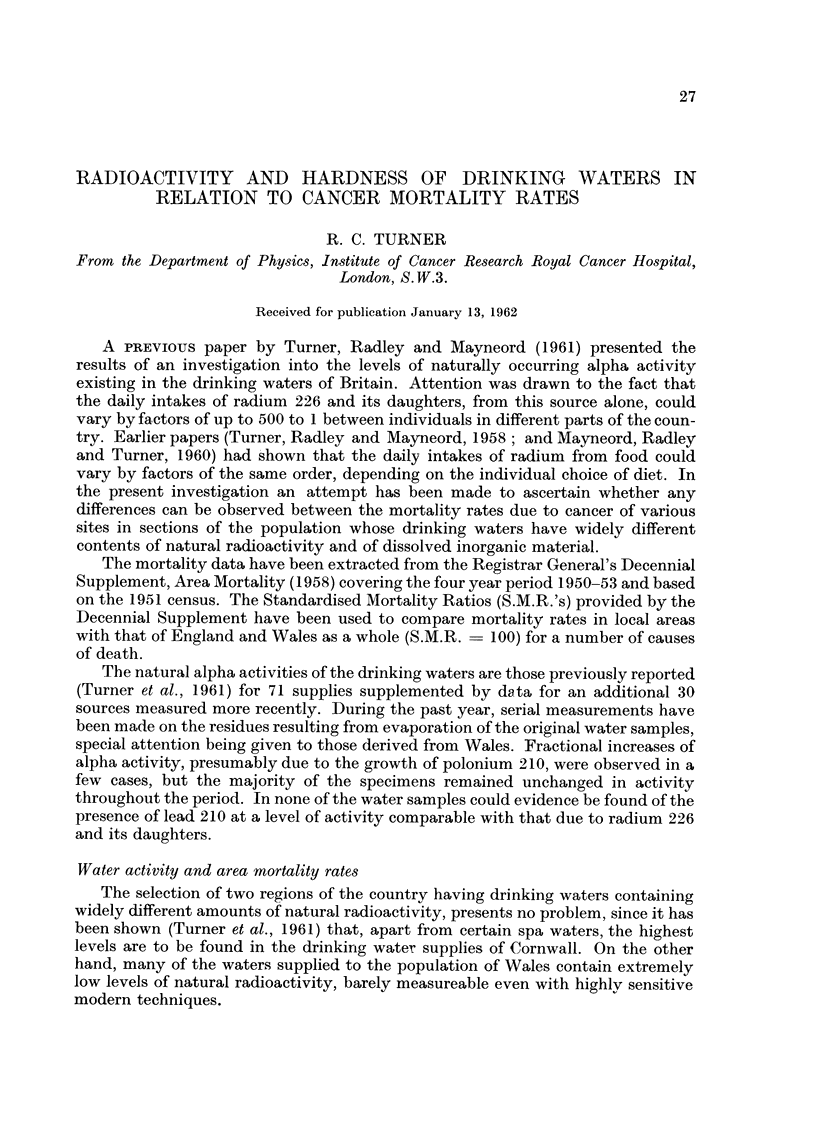

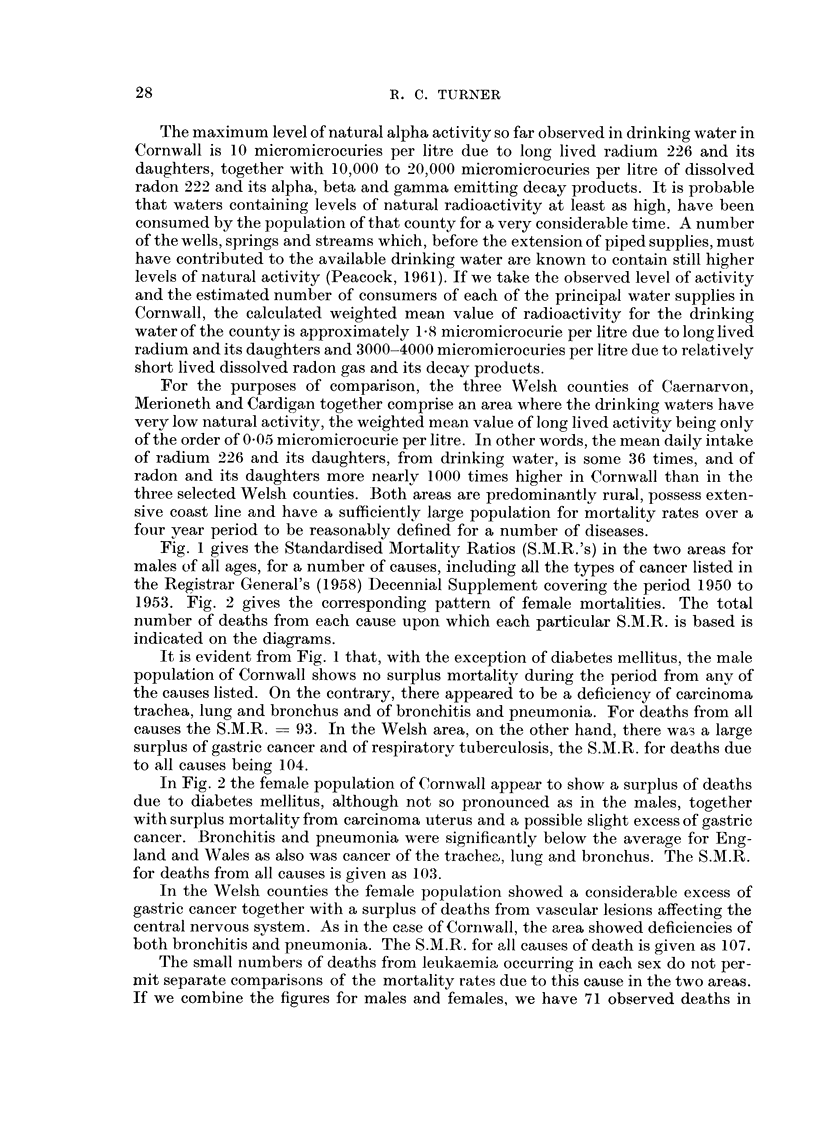

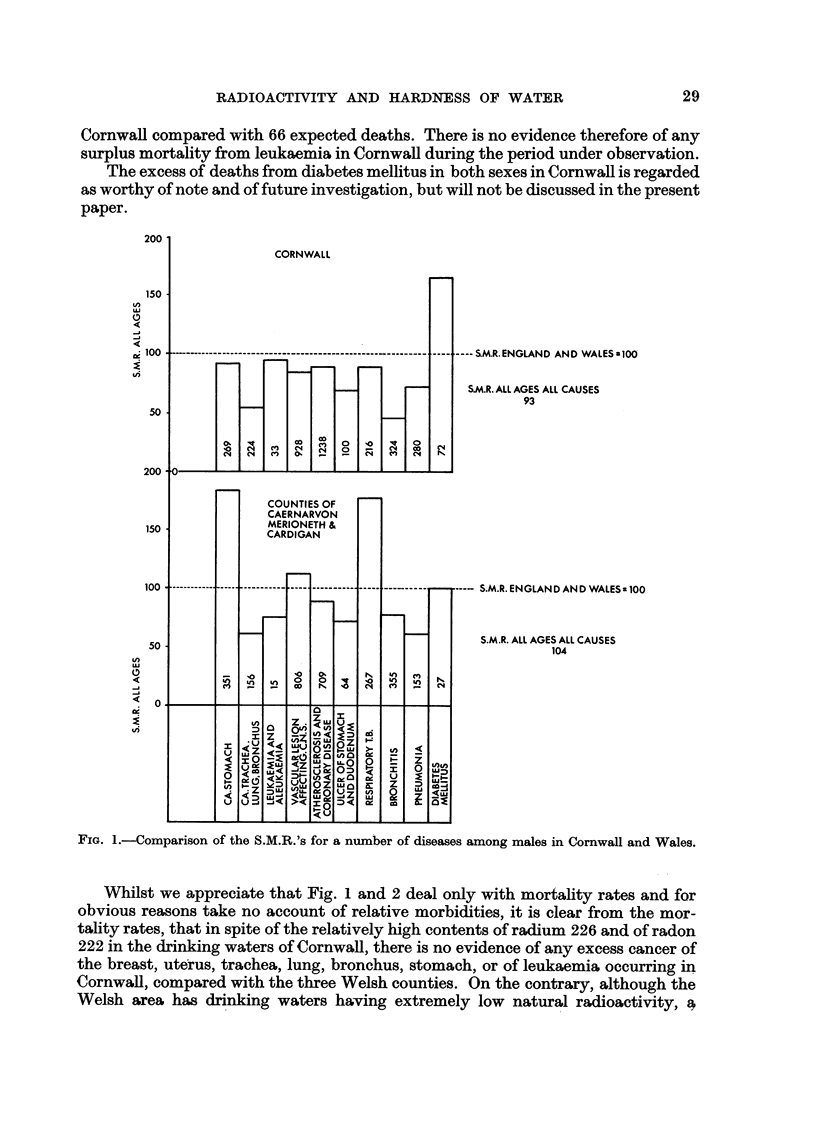

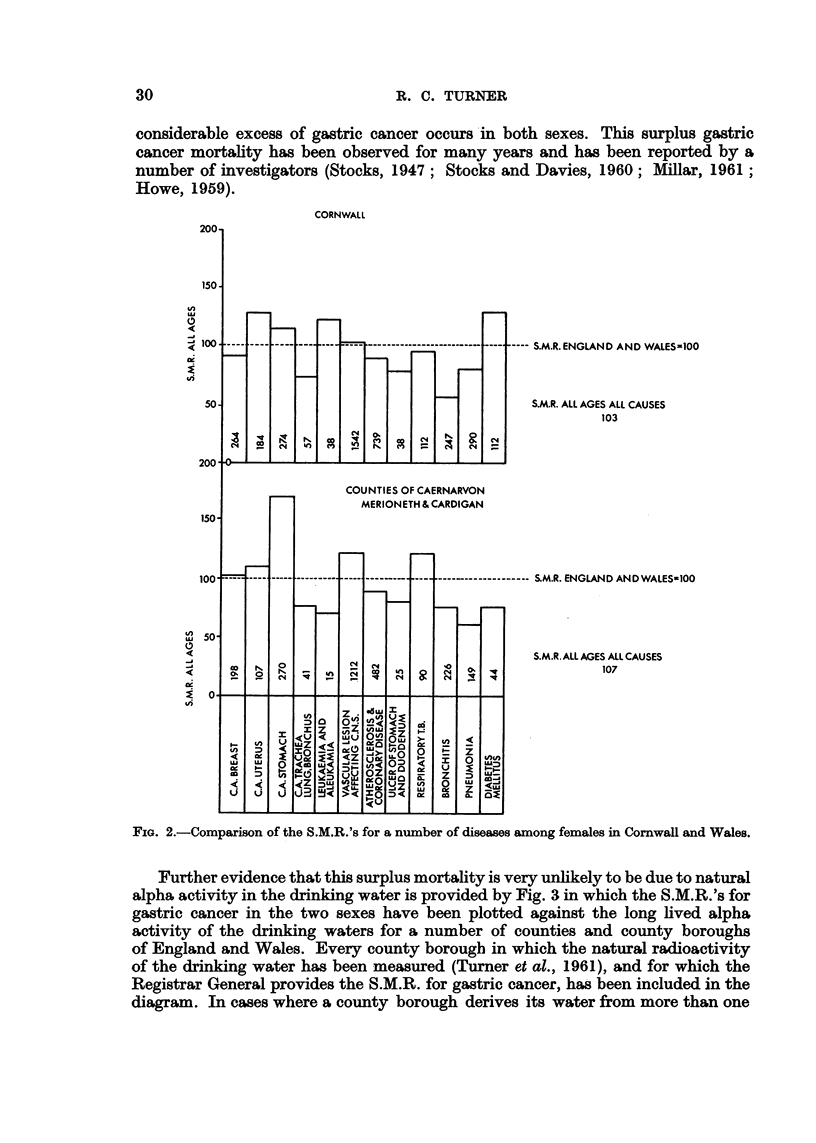

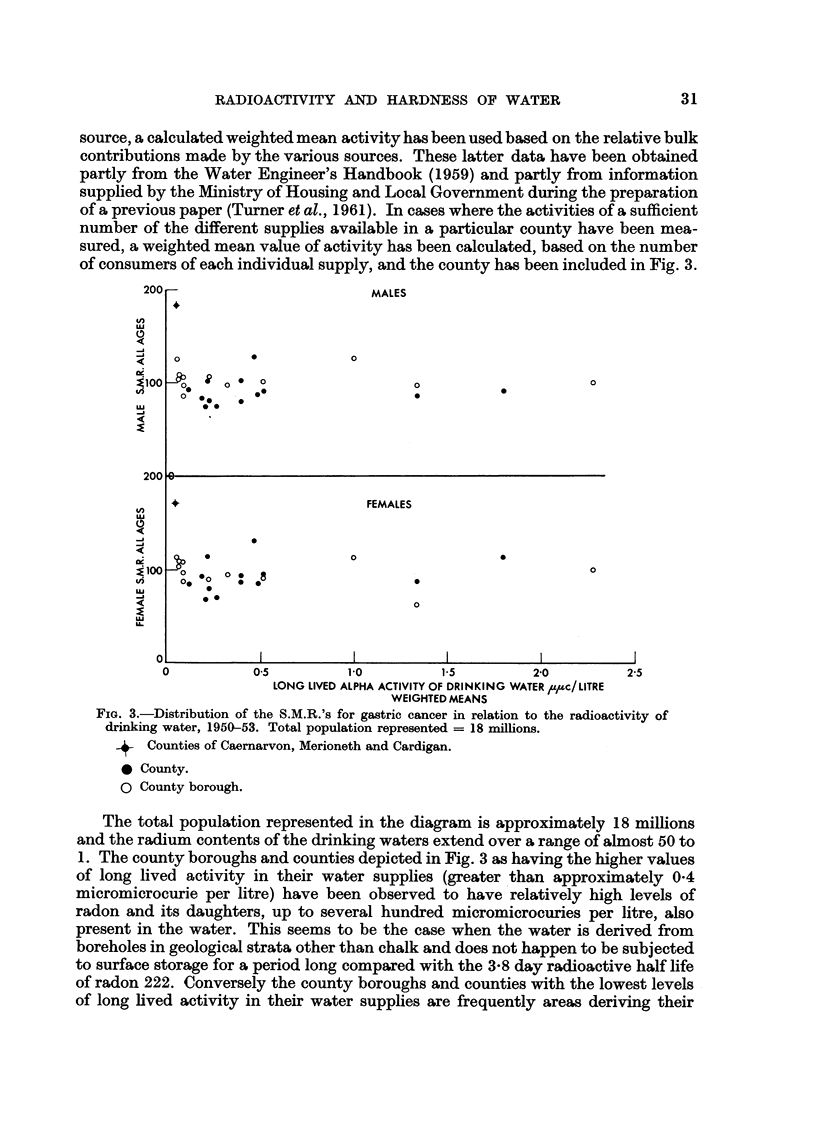

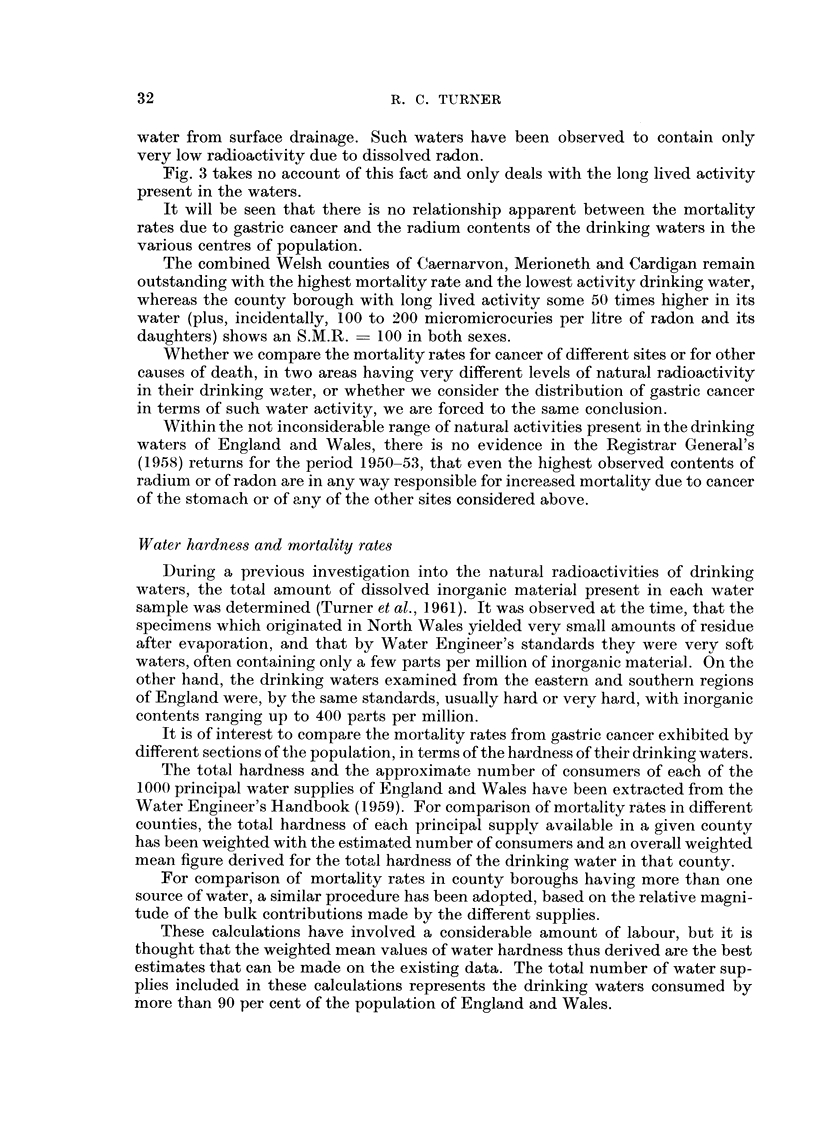

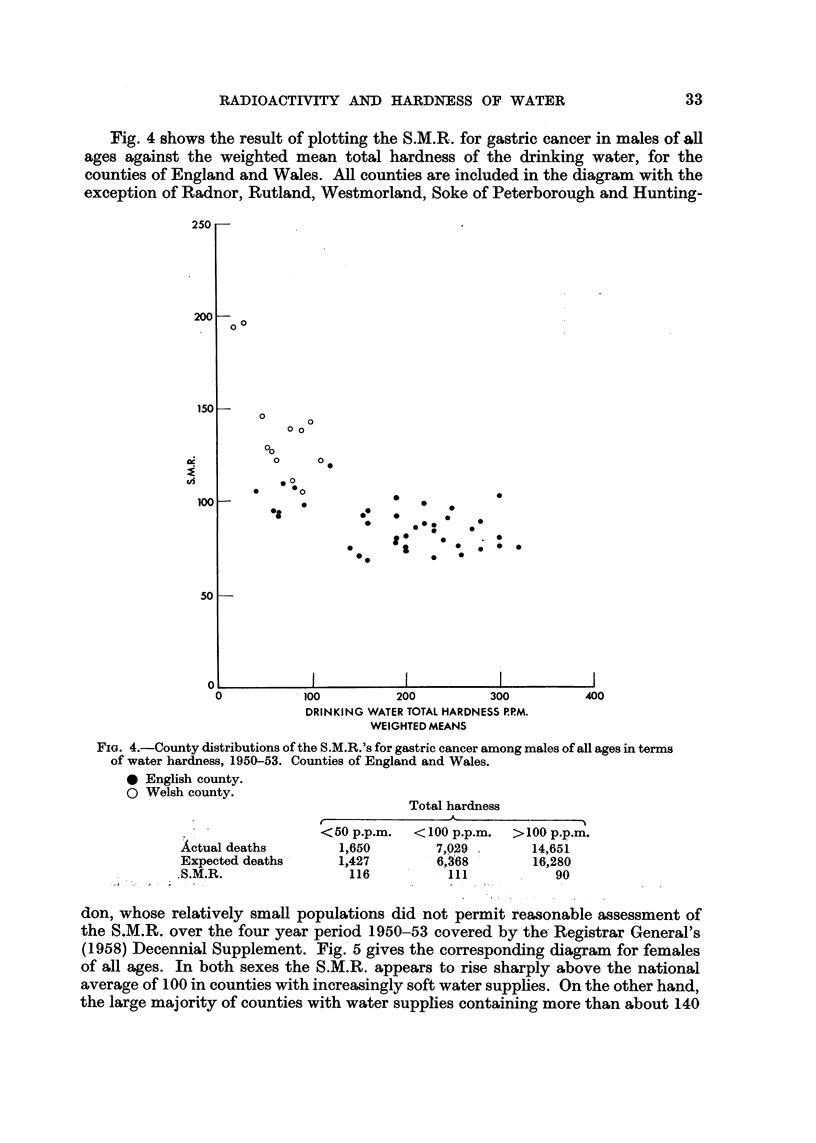

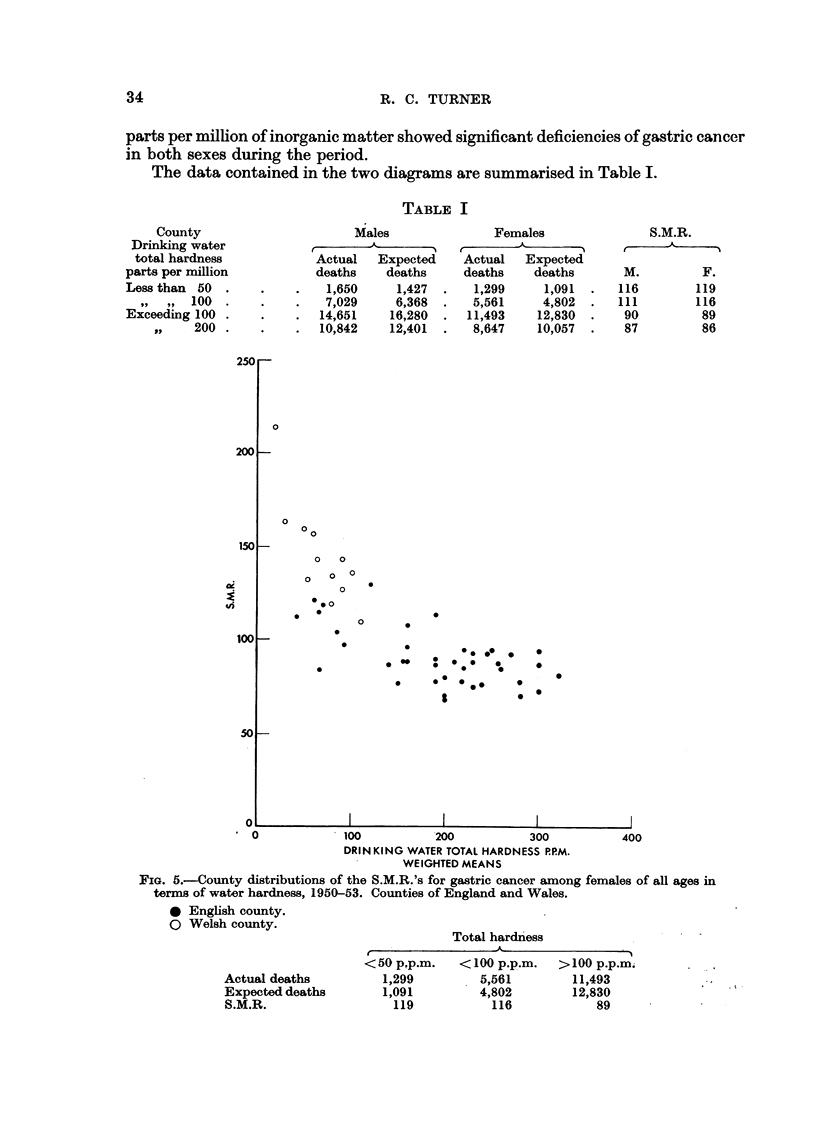

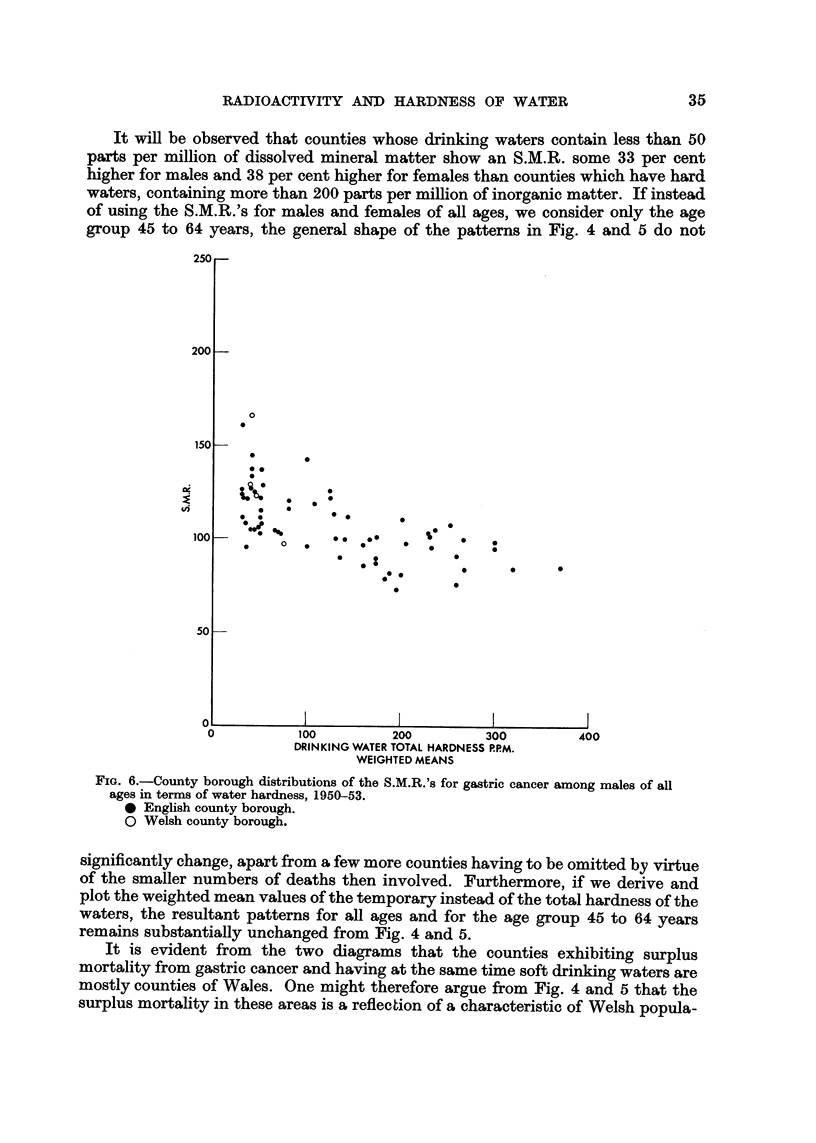

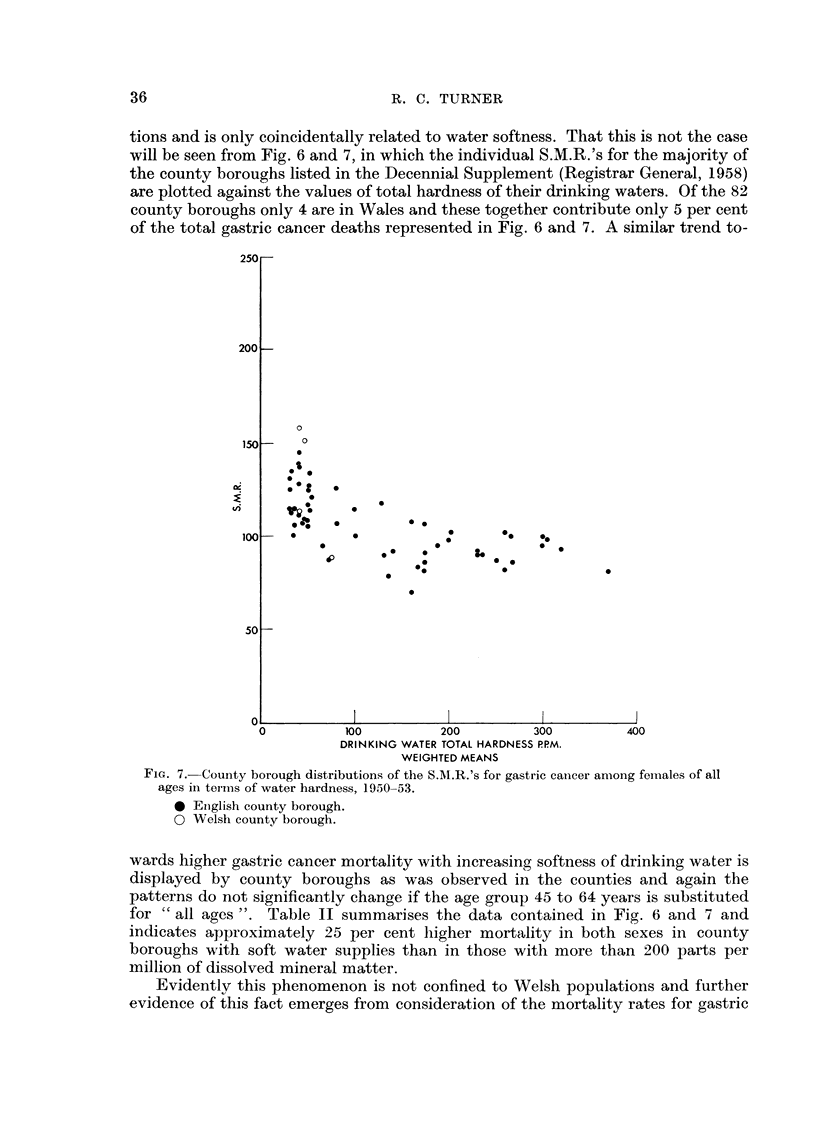

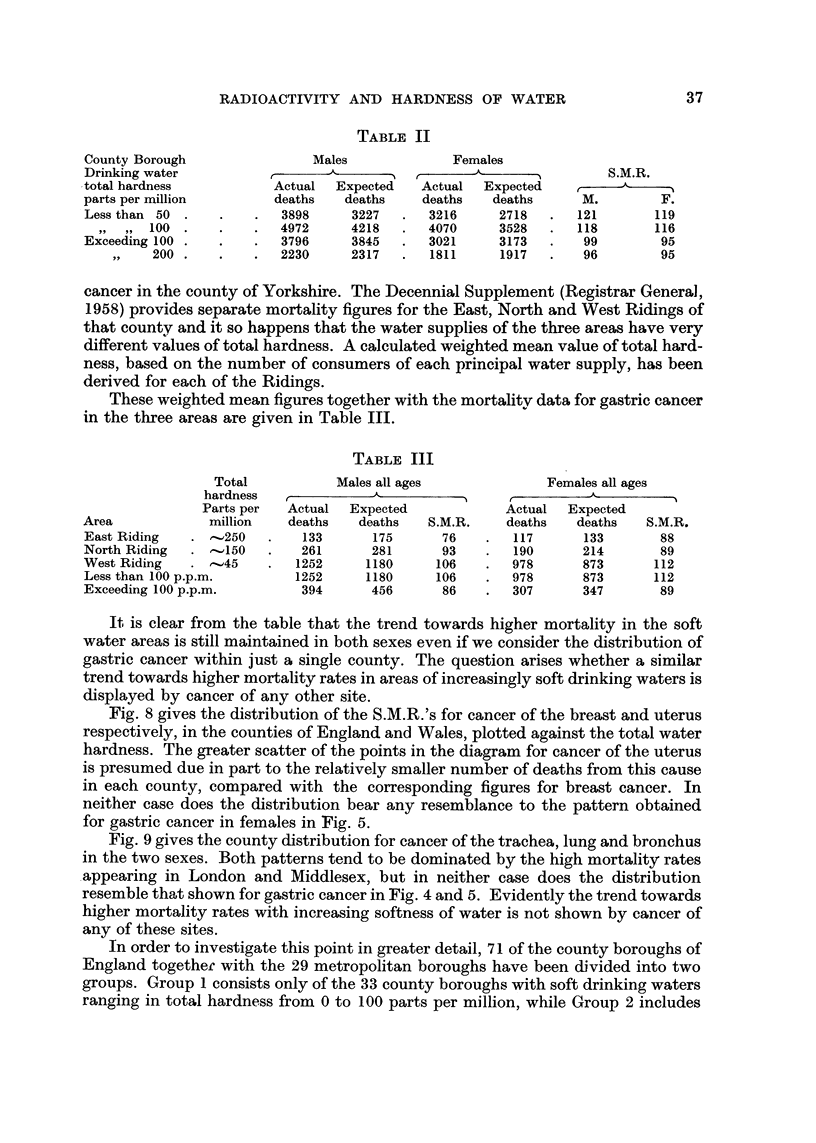

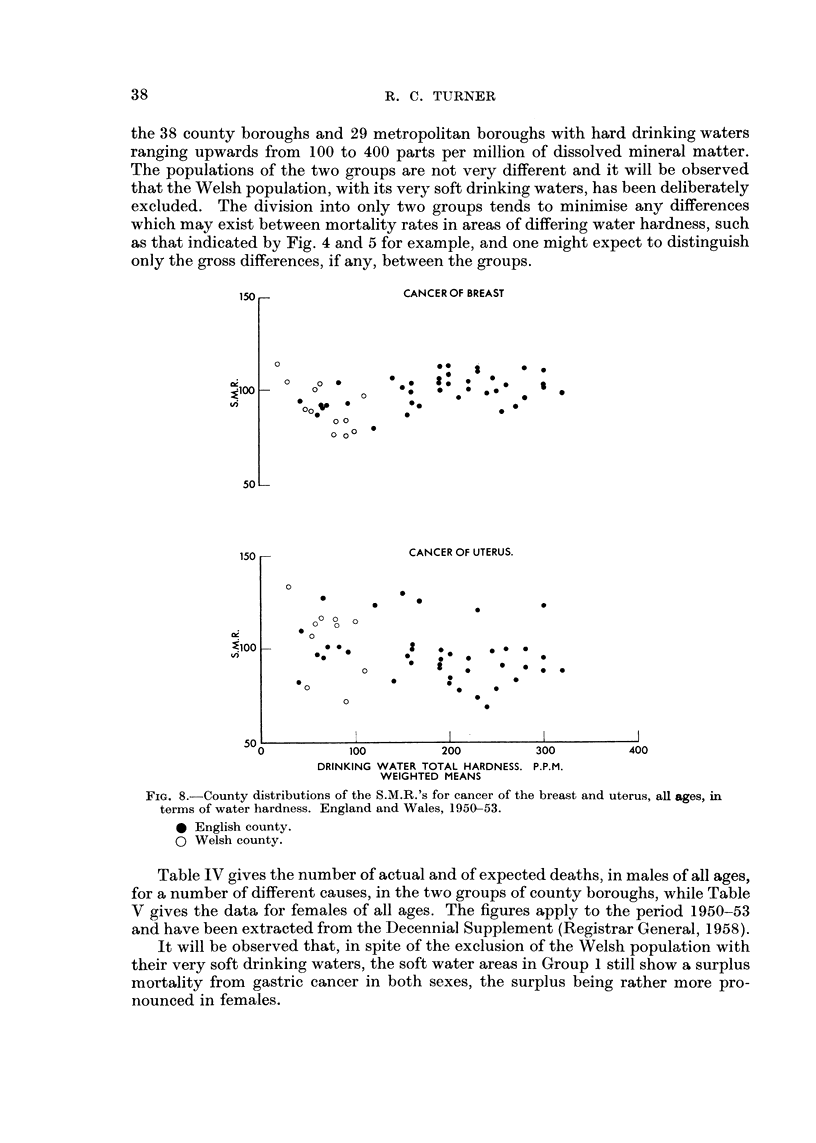

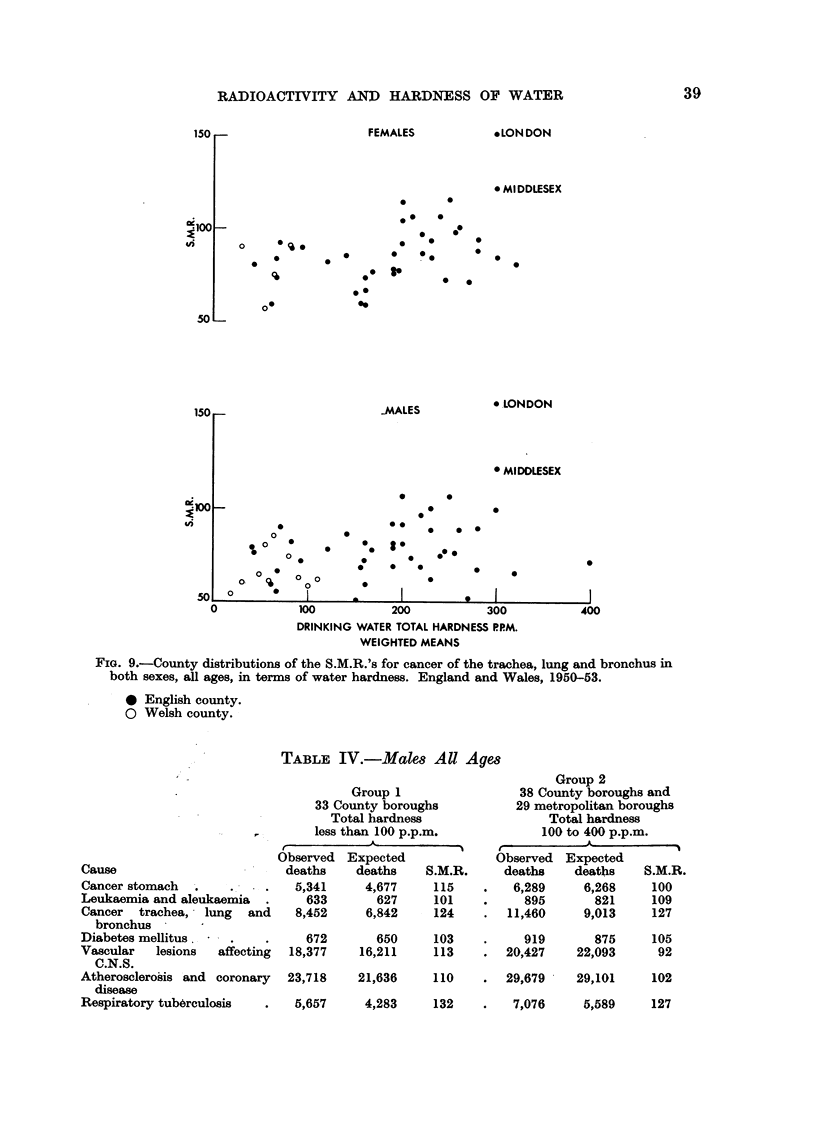

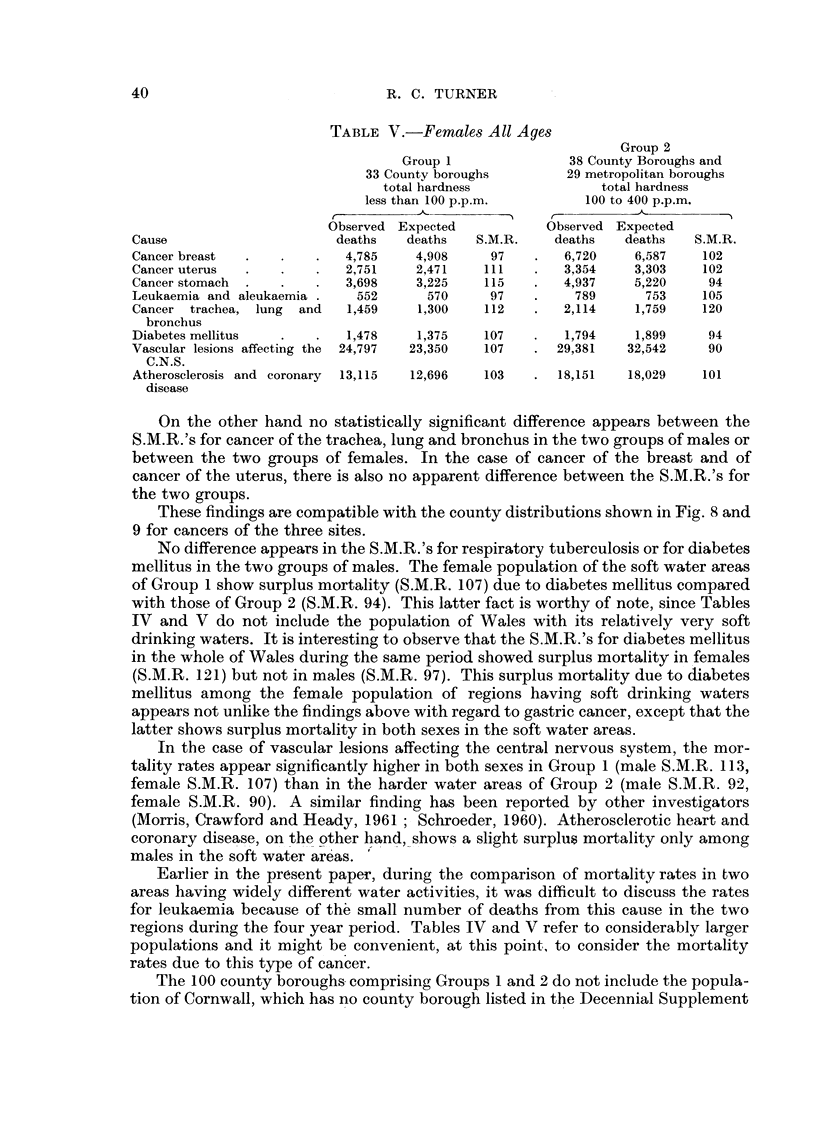

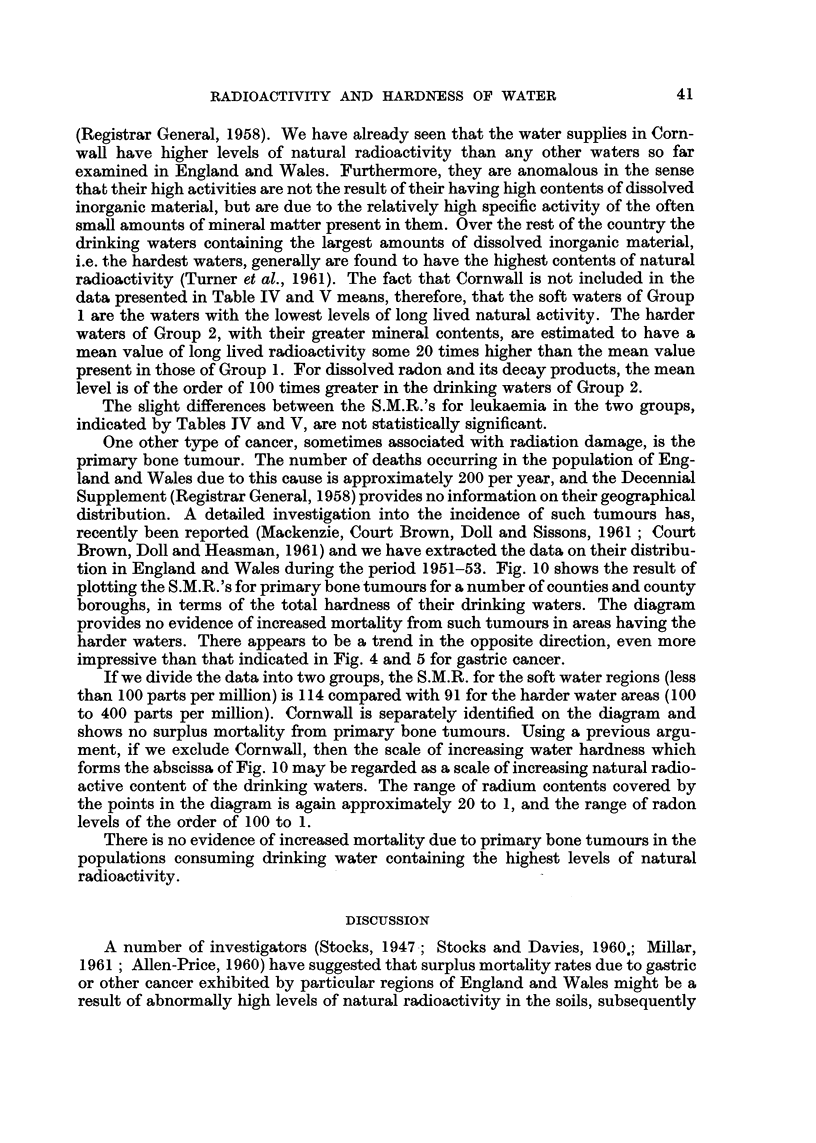

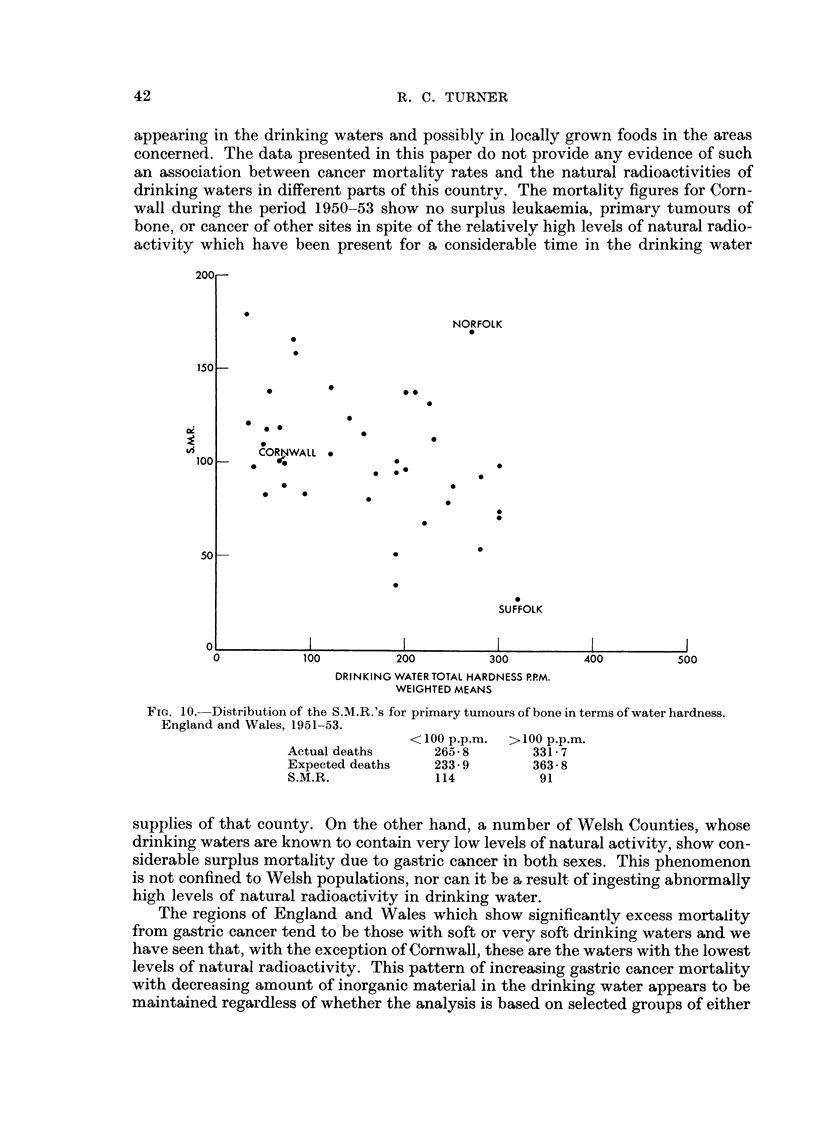

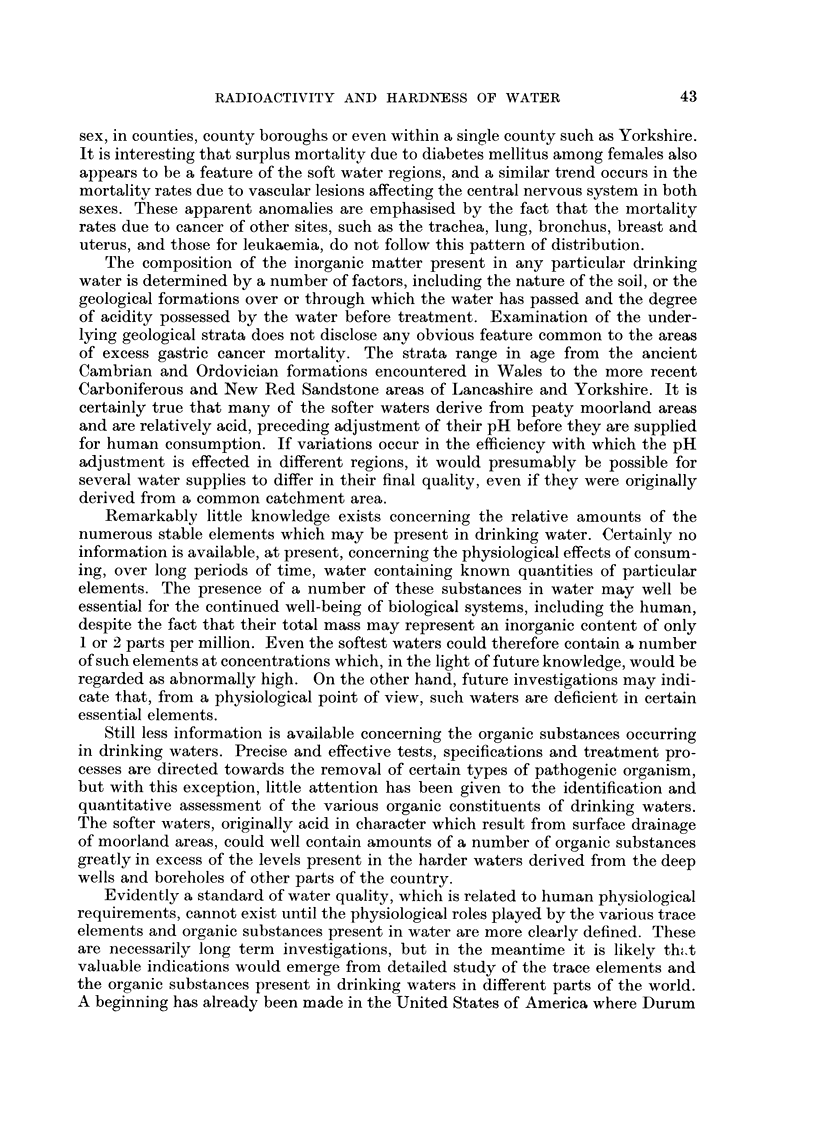

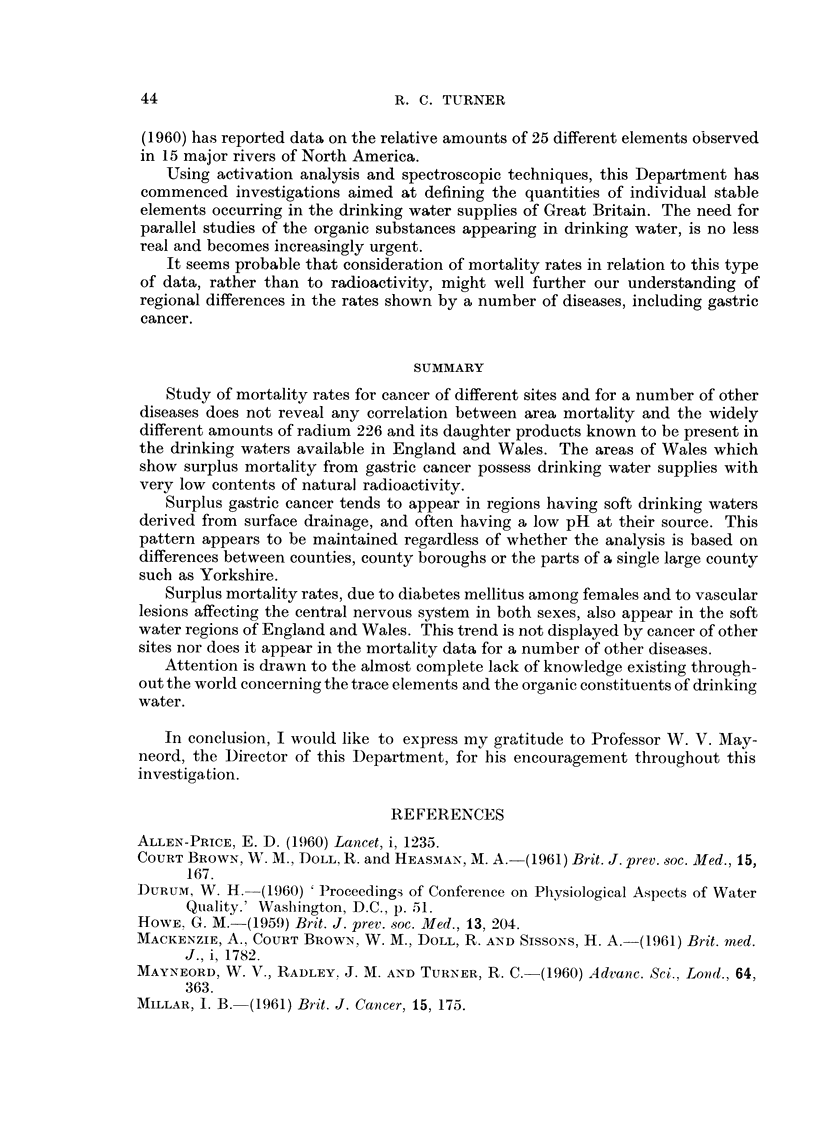

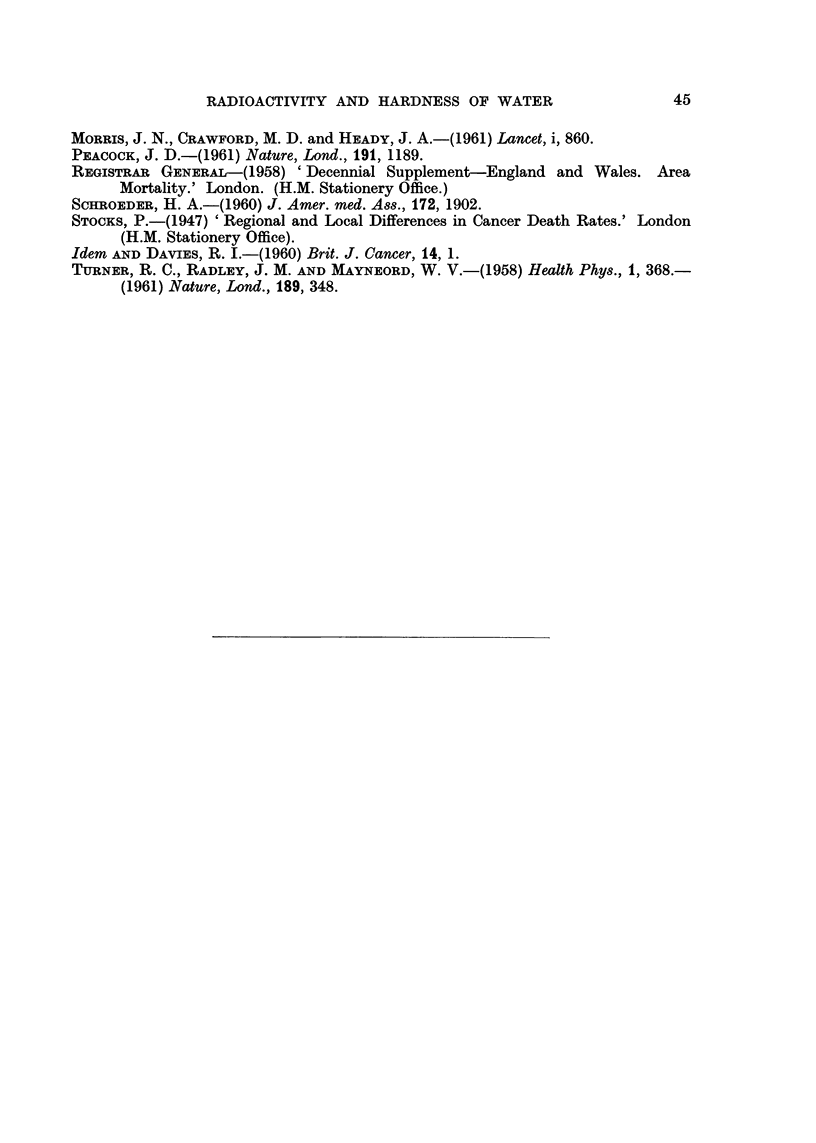


## References

[OCR_01751] MORRIS J. N., CRAWFORD M. D., HEADY J. A. (1961). Hardness of local water-supplies and mortality from cardiovascular disease in the County Boroughs of England and Wales.. Lancet.

[OCR_01765] TURNER R. C., RADLEY J. M., MAYNEORD W. V. (1961). Naturally occurring alpha-activity of drinking waters.. Nature.

